# Changes in Subfoveal Choroidal Thickness Following Uncomplicated Cataract Surgery: A Scoping Review

**DOI:** 10.7759/cureus.40861

**Published:** 2023-06-23

**Authors:** Konstadinos Sotiropulos, Dimitrios Kourkoutas, Klio I Chatzistefanou, Konstantinos Droutsas, Marilita M Moschos

**Affiliations:** 1 Department of Ophthalmology, 417 Army Shared Fund Hospital, Athens, GRC; 2 Department of Ophthalmology, 401 General Military Hospital of Athens, Athens, GRC; 3 1st Department of Ophthalmology, University of Athens, Athens, GRC

**Keywords:** uncomplicated cataract surgery, scoping review, optical coherence tomography angiography, choroidal thickness, subfoveal choroidal thickness changes

## Abstract

This scoping review investigates the relationship between subfoveal choroidal thickness (SFCT) and cataract surgery. We synthesized existing research to clarify SFCT changes following surgery and identify contributing factors. Our aim was to enhance understanding of the ocular changes associated with cataract surgery.

A comprehensive database search identified studies on SFCT changes after uncomplicated cataract surgery, categorized as “SFCT changes after cataract surgery,” “Factors influencing SFCT,” “Macular thickness changes,” and “Long-term changes.” Quantitative data and findings were extracted from a total of 13 research articles.

Studies on SFCT changes after cataract surgery provided valuable insights for the subject under review. Factors influencing SFCT changes included age, axial length, and cataract surgery. Macular thickness increased at various time points. Long-term SFCT changes varied among studies.

The present research provides valuable insights into SFCT changes after cataract surgery. Factors including age, axial length, and cataract surgery consistently influence SFCT. The clinical relevance of macular thickness changes remains uncertain, and the influence of age, ocular pathologies, and patient populations is highlighted. Long-term changes in SFCT vary, suggesting a need for further research. Standardized measurement techniques and larger cohort studies are recommended to enhance comparability and generalizability. This review enhances understanding of ocular changes associated with cataract surgery and informs future research.

## Introduction and background

Choroid plays a vital role in homeostasis, nutrient delivery, and volume maintenance in the eye [1]. Choroidal thickness has been shown to have an association with various ocular pathologies and surgical interventions [2-4]. Choroidal thickness is a commonly studied parameter [5], along with other choroidal biomarkers such as choroidal volume, choroidal vessel layer thickness, and the choroidal vascularity index. Choroidal thickness is conventionally measured on optical coherence tomography (OCT) from the hyperreflective posterior edge of the retinal pigment epithelium (RPE) to the choroidoscleral interface where the reflectivity changes [6].

However, the data on the change in choroidal thickness after phacoemulsification cataract surgery are not clear. Some studies have reported an increase [4], whereas others have reported no change in choroidal thickness [7]. Recent advancements in choroidal imaging techniques, including enhanced depth imaging optical coherence tomography, swept-source optical coherence tomography, en-face optical coherence tomography, and optical coherence tomography angiography have facilitated in-depth analysis of the choroid. Therefore, we performed this review of the available published literature to investigate the relationship between SFCT and cataract surgery.

The choroid, a vascular layer located between the retina and the sclera, plays a vital role in maintaining homeostasis, delivering nutrients, and regulating the volume within the eye [1]. Choroidal thickness, a parameter frequently studied in ocular research, has been found to have associations with various ocular pathologies and surgical interventions [2-4]. Additionally, choroidal thickness is conventionally measured using optical coherence tomography (OCT) by measuring the distance from the hyperreflective posterior edge of the RPE to the choroidoscleral interface where the reflectivity changes [6].

Previous studies have investigated the relationship between choroidal thickness and cataract surgery, but the findings have been inconsistent. Some studies have reported an increase in choroidal thickness following phacoemulsification cataract surgery [4], while others have found no significant change [7]. This discrepancy may be attributed to various factors, including differences in study design, sample sizes, and methodologies.

Advancements in choroidal imaging techniques have provided new opportunities for in-depth analysis of the choroid. Enhanced depth imaging optical coherence tomography, swept-source optical coherence tomography, en-face optical coherence tomography, and optical coherence tomography angiography are among the innovative imaging modalities that have facilitated a more comprehensive evaluation of the choroidal structure and vascular dynamics.

Given the inconsistent findings regarding choroidal thickness changes after phacoemulsification cataract surgery and the recent advancements in choroidal imaging techniques, it is crucial to conduct a systematic review of the available literature to gain a better understanding of the relationship between subfoveal choroidal thickness (SFCT) and cataract surgery.

The aim of this paper is to conduct a scoping review of the available literature to investigate the relationship between SFCT and cataract surgery. By synthesizing existing research, we aim to clarify the current understanding of how choroidal thickness may change following cataract surgery and identify potential factors contributing to the inconsistent findings reported in previous studies. This review will provide valuable insights into the relationship between SFCT and cataract surgery, contributing to a better understanding of the ocular changes associated with this surgical intervention.

## Review

Methods

The current study was conducted as a scoping review [8,9]. We adopted the five-stage framework of Arksey and O’Malley (2005, p. 22) as follows: (1) identifying research questions, (2) identifying relevant articles, (3) study selection, (4) charting the data, and (5) collating, summarizing, and reporting the results.

The following five steps were applied.

Identifying Research Questions

We aim to answer the following two questions.

(1) what is the influence of phacoemulsification on SFCT in patients undergoing uncomplicated cataract surgery?

(2) What are the reported factors contributing to choroidal thickness changes among surgical patients undergoing uncomplicated cataract surgery?

Identifying Relevant Studies

The search terms were developed and categorized based on three dimensions according to the purpose of the review. One dimension was related to choroid (i.e., the tissue examined), the second dimension was related to OCT (i.e., the method used to assess choroid) while the third dimension was related to cataract surgery (i.e., the type of surgery eyes underwent) to narrow the search within the field of operated eyes. Each search term was separated by the Boolean OR operator and each dimension was separated by the Boolean AND operator, as follows: (“Choroid” OR “Subfoveal Choroidal Thickness” OR “Fovea”) AND (“Optical coherence tomography”) AND (“Uncomplicated cataract surgery” OR “Uncomplicated phacoemulsification” OR “Phacoemulsification”).

Two electronic databases were searched - Pubmed and Scopus. Moreover, a manual search or “hand-searching” [10] reference lists was conducted after searching the databases to locate relevant studies missing in the database searches. This scoping review included primary research studies of any design and systematic reviews from any country that reports on SFCT following cataract surgery.

Inclusion and exclusion criteria: The inclusion criteria were as follows: (1) all studies that were published until September 30, 2022, (2) studies recruiting patients who underwent cataract extraction and intraocular lens implant (phacoemulsification); (3) SFCT before and after cataract surgery was measured, (4) patients included in the studies should not exhibit comorbidity. The exclusion criteria were as follows: (1) articles published in any language other than English, (2) articles not peer-reviewed (usually preprints and abstracts from conferences, editorials), and (3) duplicate publications were excluded.

Study Selection

**Figure 1:**
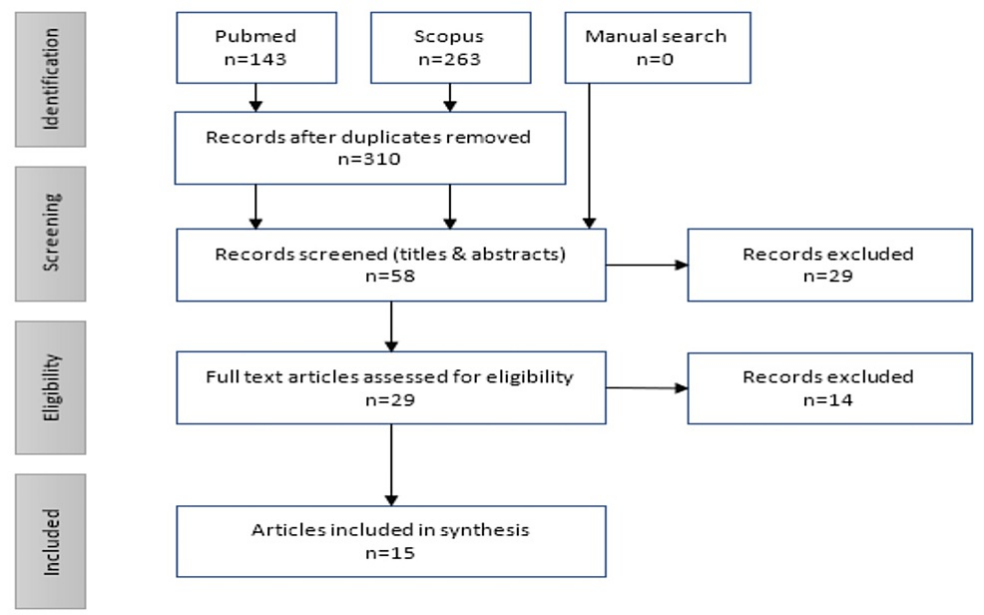
Study flow diagram (PRISMA-ScR flow chart)

The three stages of study selection were based on title, abstract, and full-text searches respectively according to the relevant sections of the Preferred Reporting Items for Systematic Reviews and Meta-Analyses Extension for Scoping Reviews (PRISMA-ScR) guideline (Figure 1) [11].

Charting the Data

Evaluating each study involved a combination of numerical description and general thematic analysis. For the former, the following information was extracted from each article: authors; country of authorship; journal title; the year of publication; study design; sample size; type of OCT used; factors affecting SFCT; quantitative outcomes. Through thematic analysis, other details about the studies were recorded, including, important author’s quotes, theoretical considerations, and any insights pertinent to the research area.

Collating, Summarizing, and Reporting the Results

The information drawn from each article was summarized and tabulated (Table 1).

**Table 1:** Information extracted from the articles

Author(s)	Country of authorship	Study Title	Journal	Year	Study design	Sample size	Type of OCT used	Parameters measured	SFCT measurement method	Factors affecting SFCT	Study findings
Noda et al. [4]	Japan	Long-term increase in subfoveal choroidal thickness after surgery for senile cataracts	American Journal of Ophthalmology	2014	Retrospective cohort study	29 patients	Spectralis SD-OCT	Central retinal thickness	Manual measurement method	Male gender and thicker baseline subfoveal choroidal thickness	Baseline subfoveal choroidal thickness was 193.8 μm, increasing significantly at postoperative 1, 3, and 6 months (208.9 μm, 210.2 μm, 209.3 μm; P < 0.0001). 69.0% maintained increased subfoveal choroidal thickness for 6 months. Male gender and thicker baseline choroid predicted larger increases.
Falcão et al. [7]	Portugal	Choroidal and macular thickness changes induced by cataract surgery	Clinical Ophthalmology (Auckland, N.Z.)	2014	Not specified, likely prospective observational study	14 patients	Enhanced Depth Imaging Spectral Domain Optical Coherence Tomography	Retinal and choroidal thickness	OCT EDI B	Cataract surgery, Use of topical steroidal and nonsteroidal anti-inflammatory drugs	Pre-surgery, retinal thickness was 308.77±14.69 μm (vertical) and 304.49±15.37 μm (horizontal). Choroidal thickness varied. Post-surgery, macular thickness significantly increased at different time points. No significant changes in choroidal thickness were observed.
Akcam et al. [12]	Turkey	Changes in choroidal and foveal retinal thickness after cataract surgery	Surgeon	2022	Prospective comparative observational study	20 persons in 2 groups	Spectral-domain OCT (SD-OCT)	Subfoveal choroidal thickness (SFCT) and foveal retinal thickness (FRT)	Enhanced depth imaging SD-OCT (EDI-OCT) with manual caliper measurements by two independent observers blinded to the clinical data	Age, gender, axial length, intraocular pressure, and other ocular and systemic factors	FT values remained unchanged, while CT increased transiently postoperatively, but returned to preoperative levels. The minimal and non-clinically significant changes in CT were supported by quantitative data.
Chen et al. [13]	China	Subfoveal Choroidal Thickness After Femtosecond Laser-Assisted Cataract Surgery for Age-Related Cataracts	Front Med (Lausanne)	2022	Prospective Study	52 patients in 2 groups	Spectral Domain Optical Coherence Tomography (SD-OCT)	SFCT and FRT changes following cataract surgery; phacoemulsification time and energy; preoperative and postoperative aqueous flare	The measurements were performed by two independent examiners using calipers and the Heidelberg Eye Explorer software	Age, AL, preoperative IOP, preoperative flare, phacoemulsification time, phacoemulsification energy, change in IOP, change in flare	The mean SFCT showed no difference with the baseline value at 1 week after Femtosecond Laser-Assisted Cataract Surgery (FLACS). However, an increased SFCT was observed in the Conventional Phacoemulsification Surgery (CPS) group. Aqueous flare was significantly minor in the FLACS group than that in the CPS group in the early postoperative stage. At 3 months post-surgery, there were no differences between the two groups.
Maul et al. [14]	Chile	Choroidal thickness measured by spectral domain optical coherence tomography: factors affecting thickness in glaucoma patients	Ophthalmology	2011	Cross-sectional study	37 glaucoma patients, 37 glaucoma suspects	Spectral Domain Optical Coherence Tomography (SD-OCT)	Axial length, CCT, and diastolic perfusion pressure, macular choroidal thickness, peripapillary choroidal thickness	Averaging choroidal thickness over the scan extension	Significant associations found between choroidal thickness and age, axial length, CCT, and diastolic ocular perfusion pressure.	The study achieved high visualization rates of the choroidal-scleral interface. Factors influencing macular choroidal thickness were identified, including eye length, age, diastolic ocular perfusion pressure, and corneal thickness. Glaucoma did not significantly affect choroidal thickness or its association with disease severity.
Gudauskiene et al. [15]	Czech Republic	Changes in subfoveal choroidal thickness after uncomplicated cataract surgery	Biomed Pap Med Fac Univ Palacky Olomouc Czech Repub	2019	Observational	30 eyes from 23 patients	Swept-source optical coherence tomography (SS-OCT)	BCVA, IOP, AL, FRT, SFCT	Using SS-OCT at 1050 nm, retinal-choroidal maps were generated for ETDRS subfields. Sector thickness was measured within specific distances from the fovea.	Age, AL, preoperative IOP, postoperative IOP, OPP	FRT significantly increased after surgery in all sectors and remained stable between 1 and 3 months. SFCT increased significantly at 3 months post-surgery. Reduced IOP led to increased OPP. Negative correlation between IOP changes and SFCT changes. Phacoemulsification induced short-term SFCT thickening. Subclinical increase in FRT resolved but didn't reach preoperative values.
Ibrahim et al. [16]	Egypt	Changes in Choroidal Thickness after Cataract Surgery	Seminars in Ophthalmology	2018	Prospective observational study	53 patients	Enhanced depth imaging-OCT (EDI-OCT)	Pre- and post-operative SFCT, subfoveal thickness, macular thickness	Manual caliper measurements using the built-in software	Age, axial length (AL), preoperative intraocular pressure (IOP), best corrected visual acuity (BCVA), and operative time	SFCT significantly increased at D7 after surgery (mean 228.42 ± 59.77 μm) and decreased to near baseline at M3 (mean 200.63 ± 61.37 μm); negative correlation between SFCT changes and IOP; negative correlation between AL and SFCT; one patient developed pseudophakic cystoid macular edema (PCME) at W6 after surgery, with highest SFCT values observed at D7 after surgery
Ohsugi et al. [17].	Japan	Changes in choroidal thickness after cataract surgery	Journal of Cataract and Refractive Surgery	2014	Prospective observational study	50 right eyes and 50 left eyes from 100 patients	Spectral-domain OCT	Choroidal thickness at 5 points	Manual tracing of the RPE and choroidal-scleral interface	Cataract type and severity; age; axial length	Postoperative choroidal thickness increased in specific regions, correlating with IOP and axial length. Absence of cystoid macular edema. Macular thickness increased significantly but not clinically relevant. Pediatric eyes had low CME incidence. SD-OCT effective for CME assessment. Larger prospective studies needed.
Sacchi et al. [18]	Italy	Spectral-domain optical coherence tomography measurements of central foveal thickness before and after cataract surgery in children	Journal of Cataract & Refractive Surgery	2015	Prospective, observational	11 patients	Spectral-domain OCT	Central foveal thickness	Manual caliper	Age, posterior capsulectomy and anterior vitrectomy	No CME detected. Significant increase in macular thickness at 1 and 3 months. Pediatric eyes have low CME incidence due to favorable factors. SD-OCT effective for CME assessment. Larger prospective studies needed.
Shahzad et al. [19]	Pakistan	Choroidal thickness changes following cataract surgery using swept source optical coherence tomography	Canadian Journal of Ophthalmology	2018	Prospective, interventional	202 eyes from 101 participants	Swept-source OCT (SS-OCT)	SFCT	In-built caliper tool	Age, diabetes status, time after surgery	SFCT gradually increased at 1 month after cataract surgery, more pronounced in younger and nondiabetic individuals
Von Jagow et al. [20]	Germany	Macular thickness after uneventful cataract surgery determined by optical coherence tomography	Graefes Arch Clin Exp Ophthalmol	2007	Prospective Study	33 patients	Time domain OCT	Measurements of mean minimal foveal thickness (MMFT) and mean foveal thickness (MFT) at multiple time points:	Preoperatively, 1 day, 1 week, and 6 weeks postoperatively. Best-corrected visual acuity (BCVA) tests and slit-lamp examinations were also performed during these visits.	Several ocular parameters including axial length, anterior chamber depth, phacotime, and energy were documented.	Postoperatively, MFT increased significantly at one day and 6 weeks. MMFT also increased significantly at one day and 6 weeks. No macular edema observed. Mild foveal thickness increase after cataract surgery.
Yilmaz et al. [21]	Turkey	Long-term changes in subfoveal choroidal thickness after cataract surgery	Medical Science Monitor	2016	Prospective observational study	65 eyes from 65 patients	SD-OCT	Subfoveal choroidal thickness (SFCT), macular thickness, Intraocular Pressure (IOP)	SCT was recorded from the subfoveal area using EDI with the same OCT. Because choroidal thickness demonstrates diurnal variation, measurement was made at the same time each day (between 09.30 and 11.00 hours)	Prostaglandins and cytokines released during surgery, changes in intraocular pressure	Preoperatively, CMT was 252.4 ± 27.6 μm, while at postoperative week 1 and months 1, 3, 6, and 12, CMT values ranged from 252.8 ± 21.7 μm to 257.4 ± 27.2 μm. SCT showed a slight non-significant increase. These findings contribute to understanding late-onset AMD after cataract surgery.
Pierru et al. [22]	France	Measurement of Subfoveal Choroidal Thickness After Cataract Surgery in Enhanced Depth Imaging Optical Coherence Tomography	Investigative Opthalmology & Visual Science	2014	Prospective study	95 patients	Enhanced Depth Imaging Optical Coherence Tomography (EDI-OCT)	Subfoveal choroidal thickness (SFCT)	SFCT measured from the outer part of the hyperreflective line corresponding to the retinal pigment epithelium (RPE) to the inner sclera.	Cataract surgery	In this study, SFCT at baseline was 224 ± 75 μm. Following cataract surgery, SFCT significantly increased at Day 1, Month 1, and Month 3. CRT also increased. Three patients developed PCME with the greatest SFCT progression after surgery. SFCT changes preceded PCME occurrence.

Results

Table 1 provides an overview of the studies included in this article. The studies conducted by Akcam et al., Maul et al., Chen et al., Falcão et al., Gudauskiene et al., Ibrahim et al., Noda et al., Ohsugi et al., Sacchi et al., Shahzad et al., Von Jagow et al., Pierru et al., and Yilmaz et al. investigated changes in SFCT and other parameters using various OCT techniques [4,7,12-22].

Akcam et al. [12] conducted a prospective study in Turkey using SD-OCT to measure SFCT and FRT changes after cataract surgery. SFCT was significantly decreased postoperatively (mean: 267.9 ± 61.6 μm) compared to preoperative measurements (mean: 287.5 ± 63.3 μm, p < 0.001), while FRT remained unchanged. Age and axial length were significant factors affecting SFCT.

Chen et al. [13] prospectively studied SFCT changes after FLACS, using SD-OCT to measure SFCT, FRT, phacoemulsification parameters, and aqueous flare. SFCT was measured by two independent examiners using calipers and the Heidelberg Eye Explorer software. No significant SFCT difference was found one-week post-FLACS, but increased SFCT was observed in the CPS group. FLACS group had a minor aqueous flare in the early postoperative stage.

In a cross-sectional study by Maul et al. [14] in Chile, SD-OCT was used to assess choroidal thickness in glaucoma patients. The choroid was found to be thicker under the fovea compared to a 3-mm wide zone centered on the fovea. Age, axial length, CCT, and diastolic perfusion pressure were associated with choroidal thickness, but no differences were observed between glaucoma damage and glaucoma suspects.

Falcão et al. [7] observed increased macular thickness after cataract surgery, but no significant changes in choroidal thickness one-month post-surgery. Gudauskiene et al. [15] examined SFCT changes after cataract surgery using SS-OCT, finding increased FRT in all sectors and increased SFCT at three months. Postoperative IOP reduction correlated negatively with SFCT changes in specific regions.

In a prospective observational study by Ibrahim et al. [16] in Egypt, SFCT changes following cataract surgery were investigated in 53 patients using EDI-OCT. Factors such as age, axial length, and preoperative intraocular pressure (IOP) were found to affect SFCT. The findings revealed a significant increase in SFCT at seven days after surgery, followed by a decrease toward baseline at three months.

Noda et al. [4] conducted a retrospective cohort study in Japan, including 29 patients, to examine the long-term increase in SFCT after cataract surgery. The SFCT was manually measured using Spectralis SD-OCT, and male gender and thicker baseline SFCT were identified as factors influencing SFCT. The study findings demonstrated a persistent increase in SFCT up to six months postoperatively.

In another prospective observational study by Ohsugi et al. [17] in Japan, choroidal thickness changes after cataract surgery were investigated in 100 patients using spectral-domain OCT. Factors such as cataract type, severity, age, and axial length affected SFCT. The findings showed an increase in choroidal thickness after surgery, particularly in the inferior region, and a negative correlation with axial length in the late postoperative period.

Sacchi et al. [18] conducted a prospective observational study in Italy, including 11 pediatric patients, to assess central foveal thickness before and after cataract surgery using spectral-domain OCT. Age, posterior capsulectomy, and anterior vitrectomy were identified as factors affecting SFCT. The study revealed a significant increase in mean macular thickness at one and three months, although the increase was not clinically relevant. The low incidence of cystoid macular edema in pediatric eyes after cataract surgery was attributed to various factors.

Shahzad et al. [19] performed a prospective interventional study in Pakistan involving 202 patients to investigate choroidal thickness changes after cataract surgery using swept-source OCT. SFCT was assessed using the in-built caliper tool, and age, diabetes status, and time after surgery were identified as influencing factors. The findings demonstrated a gradual increase in SFCT at one-month post-surgery, with a more pronounced increase observed in younger and nondiabetic individuals.

Von Jagow et al. [20] conducted a prospective study in Germany, including 33 patients, to evaluate foveal thickness alterations after cataract surgery. In this study, the researchers evaluated the mean minimal foveal thickness (MMFT) and mean foveal thickness (MFT) at various time points: preoperatively, at one day, one week, and six weeks postoperatively. Alongside these measurements, assessments of best-corrected visual acuity (BCVA) and slit-lamp examination were conducted. The results revealed a mild increase in foveal thickness following cataract surgery, but this increase did not have any discernible impact on visual acuity. The observed increase in foveal thickness could be attributed to both subclinical changes and the influence of alterations in media opacity, which may affect the accuracy of the measurement technique. Notably, the study did not establish any correlation between surgical and biometric parameters, such as phacotime, energy, axial length, and the degree of macular thickening. These findings suggest that factors related to the surgical procedure and individual biometric characteristics may not contribute significantly to the observed changes in macular thickness. Further research is warranted to elucidate the underlying mechanisms and clinical implications of the observed foveal thickening after cataract surgery.

Discussion

Studies Showing SFCT Changes After Cataract Surgery

In summary, the articles in the category of "SFCT changes after cataract surgery" provide valuable insights into the alterations in SFCT following cataract surgery. Akcam et al. [12] reported a significant decrease in SFCT after surgery, while Chen et al. [13] observed no change in SFCT one week after Femtosecond Laser-Assisted Cataract Surgery (FLACS) but an increased SFCT in the Conventional Phacoemulsification Surgery (CPS) group. Gudauskiene et al. [15] demonstrated a significant increase in SFCT three months after cataract surgery, along with changes in IOP and OPP. These findings highlight the complex interplay between surgical techniques, ocular parameters, and postoperative changes in choroidal thickness.

Factors Influencing SFCT

The findings from these studies provide valuable insights into factors influencing SFCT. Age, axial length, and cataract surgery were consistently identified as factors affecting SFCT changes, indicating their significant impact on choroidal thickness. This consistency across the studies strengthens the evidence supporting the role of these factors. The examination of diastolic perfusion pressure, central corneal thickness (CCT), IOP, BCVA, operative time, and cataract type and severity in some studies expands our understanding of the multifactorial nature of SFCT changes. These findings highlight the potential influence of various physiological and surgical factors on SFCT.

Macular Thickness Changes

The findings from the studies in the “Macular thickness changes” category provide important insights into the effects of cataract surgery on macular thickness. Falcão et al. [7] reported a significant increase in mean macular thickness one week and one month after cataract surgery. Sacchi et al. [18] found significant increases in mean macular thickness at one and three months after surgery, although they noted that these changes were not clinically relevant. Von Jagow et al. [20] examined foveal thickness alterations post-cataract surgery and observed a statistically significant increase in macular foveal thickness in the first and third postoperative months. These findings indicate a potential response to the surgical intervention in terms of increased macular thickness.

However, the clinical relevance of these changes remains uncertain, as some studies mention that the observed increases may not be considered clinically significant. This suggests that the magnitude of the change may not have a substantial impact on visual function or patient outcomes. Additionally, the studies highlight the influence of factors such as age, ocular pathologies, and specific patient populations on macular thickness changes. These factors may contribute to variations in the extent and duration of macular thickness alterations observed after cataract surgery.

Long-Term Changes

The studies in the “Long-term changes” category provide valuable insights into the long-term effects of cataract surgery on SFCT. Noda et al. [4] conducted a retrospective cohort study and observed a significant increase in SFCT after cataract surgery, which persisted up to six months postoperatively. Yilmaz et al. [21] conducted a prospective observational study and observed a continuous increase in choroidal thickness during a 12-month follow-up period, although the increase was not statistically significant. They also noted a slight increase in macular thickness in the first three months after surgery, which returned to pre-surgery baseline values by month six. No cystoid macular edema was observed in any cases during the follow-up. Pierru et al. [22] performed a quantitative comparative study and found a significant increase in SFCT one month after surgery. They reported a continuous increase in SFCT until six months post-surgery, followed by a gradual decrease toward preoperative levels by 12 months.

These findings suggest that cataract surgery has a long-term impact on SFCT, with a significant increase observed in the early postoperative period. However, the subsequent changes in choroidal thickness vary among the studies. Yilmaz et al. [21] found no statistically significant difference in choroidal thickness beyond the initial increase, while Noda et al. [4] and Pierru et al. [22] reported a gradual decrease in preoperative levels. It is important to note that factors such as gender, baseline choroidal thickness, and diurnal variation may influence these long-term changes. Additionally, the slight increase in macular thickness observed in the first three months after surgery suggests a transient effect on the macula.

Limitations

The authors of the present scoping review acknowledge limitations including potential bias in the selection of articles due to specific database retrieval, potential exclusion of relevant studies from other databases or unpublished literature, limited generalizability to patients with comorbidities, variations in study quality and methodology, language bias from focusing on English-language publications, and limitations in data availability, reporting methods, and outcome measurements that may impact the ability to conduct a robust meta-analysis or draw definitive conclusions.

Recommendations

Standardized Measurement Techniques

To enhance the comparability and reliability of future studies investigating SFCT changes after cataract surgery, it is recommended to adopt standardized measurement techniques. Consistency in the use of specific OCT devices and measurement methods, such as manual caliper measurements or automated algorithms, will improve the accuracy and reproducibility of SFCT assessments.

1. The existing studies in the “SFCT changes after cataract surgery” category have relatively small sample sizes. Conducting larger cohort studies with diverse patient populations will provide more robust and generalizable evidence on the factors influencing SFCT changes. Including patients with different cataract types, severity, and ocular comorbidities will enable a more comprehensive understanding of the impact of these factors on SFCT.

2. Given the limited number of studies on long-term SFCT changes after cataract surgery, there is a need for research with extended follow-up periods. Longitudinal studies spanning several years will elucidate the duration of SFCT alterations and whether these changes persist or regress over time. Assessing SFCT at various time points beyond six to 12 months post-surgery will provide valuable insights into the long-term stability of SFCT.

3. Considering the multifactorial nature of SFCT changes, future studies should employ multivariate analysis to assess the combined effects of various factors on SFCT alterations. Analyzing the interactions between age, axial length, cataract type and severity, and surgical techniques will help delineate their contributions to SFCT changes and identify potential synergistic effects.

Incorporating these recommendations into future research endeavors will advance our understanding of SFCT and macular thickness changes following cataract surgery, contributing to improved patient care and visual outcomes.

## Conclusions

The studies included in various categories have provided valuable insights into the changes in SFCT and macular thickness following cataract surgery. The findings highlight the influence of surgical techniques, ocular parameters, and various factors on SFCT changes. Age, axial length, and cataract surgery consistently affect SFCT, while additional factors such as diastolic perfusion pressure, CCT, IOP, BCVA, operative time, and cataract type contribute to the multifactorial nature of SFCT changes.

Cataract surgery leads to a significant increase in macular thickness in the early postoperative period, but the clinical significance of these changes remains uncertain. Factors such as age, ocular pathologies, and specific patient populations influence the extent and duration of macular thickness alterations. Long-term changes in SFCT after cataract surgery show both persistent increases and regression toward preoperative levels. Monitoring SFCT over an extended period may provide insights into persistent or progressive alterations. Standardized measurement techniques, larger cohort studies, and longer follow-up periods are needed to advance our understanding of SFCT and macular thickness changes, improving patient care and visual outcomes.
